# learnMET: an R package to apply machine learning methods for genomic prediction using multi-environment trial data

**DOI:** 10.1093/g3journal/jkac226

**Published:** 2022-09-19

**Authors:** Cathy C Westhues, Henner Simianer, Timothy M Beissinger

**Affiliations:** Division of Plant Breeding Methodology, Department of Crop Sciences, University of Goettingen, 37075 Goettingen, Germany; Center for Integrated Breeding Research, University of Goettingen, 37075 Goettingen, Germany; Center for Integrated Breeding Research, University of Goettingen, 37075 Goettingen, Germany; Animal Breeding and Genetics Group, Department of Animal Sciences, University of Gottingen, 37075 Gottingen, Germany; Division of Plant Breeding Methodology, Department of Crop Sciences, University of Goettingen, 37075 Goettingen, Germany; Center for Integrated Breeding Research, University of Goettingen, 37075 Goettingen, Germany

**Keywords:** multienvironment trials, machine learning, genotype ×, environment interaction, genomic prediction, R software

## Abstract

We introduce the R-package *learnMET*, developed as a flexible framework to enable a collection of analyses on multi-environment trial breeding data with machine learning-based models. *learnMET* allows the combination of genomic information with environmental data such as climate and/or soil characteristics. Notably, the package offers the possibility of incorporating weather data from field weather stations, or to retrieve global meteorological datasets from a NASA database. Daily weather data can be aggregated over specific periods of time based on naive (for instance, nonoverlapping 10-day windows) or phenological approaches. Different machine learning methods for genomic prediction are implemented, including gradient-boosted decision trees, random forests, stacked ensemble models, and multilayer perceptrons. These prediction models can be evaluated via a collection of cross-validation schemes that mimic typical scenarios encountered by plant breeders working with multi-environment trial experimental data in a user-friendly way. The package is published under an MIT license and accessible on GitHub.

## Introduction

Large amounts of data from various sources (phenotypic records from field trials, genomic or omics data, environmental information) are regularly gathered as part of multi-environment trials (MET). The efficient exploitation of these extensive datasets has become of utmost interest for breeders to address essentially two objectives: (1) accurately predicting genotype performance in future environments; (2) untangling complex relationships between genetic markers, environmental covariables (ECs), and phenotypes to better understand the pervasive phenomenon of genotype-by-environment (G × E) interaction. 

Many R packages have recently been developed that allow to implement genomic prediction models accounting for G × E effects using mixed models: BGLR (Pérez and de Los Campos 2014), sommer (Covarrubias-Pazaran 2016), Bayesian Genomic Genotype × Environment Interaction (BGGE) (Granato *et al.* 2018), Bayesian Multi-Trait Multi-Environment for Genomic Selection (BMTME) (Montesinos-López *et al.* 2019), bWGR (Xavier *et al.* 2019), EnvRtype (Costa-Neto, Galli, *et al.* 2021), and MegaLMM (Runcie *et al.* 2021). BGGE presents a speed advantage compared to BGLR, that is explained by the use of an optimization procedure for sparse covariance matrices, while BMTME additionally exploits the genetic correlation among traits and environments to build linear G × E models. EnvRtype further widens the range of opportunities in Bayesian kernel models with the possibility to use nonlinear arc-cosine kernels aiming at reproducing a deep learning approach (Cuevas *et al.* 2019; Costa-Neto, Fritsche-Neto, *et al.* 2021), and to harness environmental data retrieved by the package.

While Bayesian approaches have been successful at dramatically improving predictive ability in multi-environment breeding experiments (Cuevas *et al.* 2017, 2019; Costa-Neto, Fritsche-Neto, *et al.* 2021), data-driven machine learning algorithms represent alternative predictive modeling techniques with increased flexibility with respect to the form of the mapping function between input and output variables. In particular, nonlinear effects including gene × gene and genotype × environment (G × E) interactions can be captured with machine learning models (Ritchie *et al.* 2003; McKinney *et al.* 2006; Crossa *et al.* 2019; Westhues *et al.* 2021). G × E interactions are of utmost interest for plant breeders, especially when they present a crossover type, because the latter implies a change in the relative ranking of genotypes across different environments. Breeders generally cope with G × E by either (1) focusing their program on wide adaptation of cultivars over a target population of environments, from which follows that the developed varieties are not the best ones for a given environment, and positive G × E interactions are not exploited, or (2) identifying varieties that are the best adapted to specific environments (Bernardo 2002). Enhancing the modeling of genotype-by-environment interactions, by the inclusion of environmental covariates related to critical developmental stages, also resulted in an increase of predictive ability in many studies using MET datasets (Heslot *et al.* 2012; Monteverde *et al.* 2019; Rincent *et al.* 2019; Costa-Neto, Fritsche-Neto, *et al.* 2021).

In this article, we describe the R-package learnMET and its principal functionalities. learnMET provides a pipeline to (1) facilitate environmental characterization and (2) evaluate and compare different types of machine learning approaches to predict quantitative traits based on relevant cross-validation (CV) schemes for MET datasets. The package offers flexibility by allowing to specify the sets of predictors to be used in predictions, and different methods to process genomic information to model genetic effects.

To validate the predictive performance of the models, different CV schemes are covered by the package, that aim at addressing concrete plant breeding prediction problems with multi-environment field experiments. We borrow the same terminology as in previous related studies (see Burgueño *et al.* 2012; Jarquín *et al.* 2014, 2017), as follows: (1) CV1: predicting the performance of newly developed genotypes (never tested in any of the environments included in the MET); (2) CV2: predicting the performance of genotypes that have been tested in some environments but not in others (also referred to as field sparse testing); (3) CV0: predicting the performance of genotypes in new environments, i.e. the environment has not been tested; and (4) CV00: predicting the performance of newly developed genotypes in new environments, i.e. both environment and genotypes have not been observed in the training set. For CV0 and CV00, four configurations are implemented: leave-one-environment-out, leave-one-site-out, leave-one-year-out, and forward prediction.

## Methods

### Installation and dependencies

Using the devtools package (Wickham *et al.* 2021), learnMET can be easily installed from GitHub and loaded (Box 1).

Box 1. Install learnMET

> devtools::install_github(“cjubin/learnMET”)

> library(learnMET)

Dependencies are automatically installed or updated when executing the command above.

### Real multi-environment trial datasets

Three toy datasets are included with the learnMET package to illustrate how input data should be provided by the user and how the different functionalities of the package can be utilized.

#### Rice datasets

The datasets were obtained from the INIA’s Rice Breeding Program (Uruguay) and were used in previous studies (Monteverde *et al.* 2018, 2019). We used phenotypic data for three traits from two breeding populations of rice (*indica*, composed of 327 elite breeding lines; and *japonica*, composed of 320 elite breeding lines). The two populations were evaluated at a single location (Treinta y Tres, Uruguay) across multiple years (2010–2012 for *indica* and 2009–2013 for *japonica*) and were genotyped using genotyping-by-sequencing (GBS) (Monteverde *et al.* 2019). ECs, characterizing three developmental stages throughout the growing season, were directly available. More details about the dataset are given in Monteverde *et al.* (2018).

#### Maize datasets

A subset of phenotypic and genotypic datasets, collected and made available by the G2F initiative (www.genomes2fields.org), were integrated into learnMET. Hybrid genotypic data were computed in silico based on the GBS data from inbred parental lines. For more information about the original datasets, please refer to AlKhalifah *et al.* (2018) and McFarland *et al.* (2020). In total, phenotypic data, collected from 22 environments covering 4 years (2014–2017) and 6 different locations in American states and Canadian provinces, are included in the package.

### Running learnMET

learnMET can be implemented as a three-step pipeline. These are described next.

#### Step 1: specifying input data and processing parameters

**Fig. 1.**
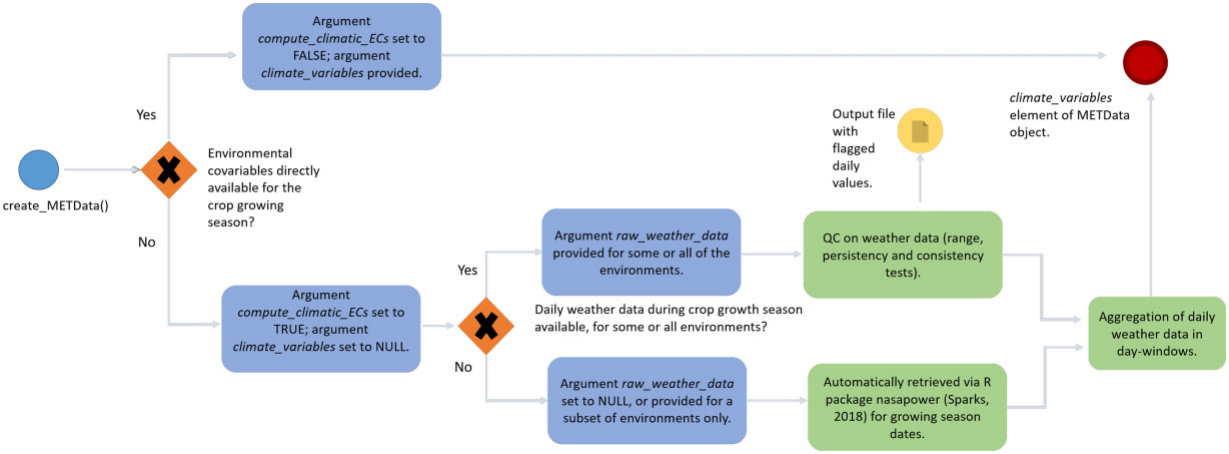
Overview of the pipeline regarding integration of weather data using the function *create_METData()* within the learnMET package. The blue circle signals the first step of the process, when the function is initially called. The blue boxes indicate how the arguments of the function should be given, according to the type of datasets available to the user. The green boxes indicate a task which is run in the pipeline via internal functions of the package. The red circle signals the final step, when the METData object is created and contains environmental covariates. Details on the quality control tests implemented on daily weather data are provided at https://cjubin.github.io/learnMET/reference/qc_raw_weather_data.html, and on the methods to build ECs based on aggregation of daily data at https://cjubin.github.io/learnMET/reference/get_ECs.html.

The first function in the learnMET pipeline is *create_METData()* (Box 2). The user must provide genotypic and phenotypic data, as well as basic information about the field experiments (e.g. longitude, latitude, planting, and harvest date). Missing genotypic data should be imputed beforehand. Climate covariables can be directly provided as day-interval-aggregated variables, using the argument *climate_variables*. Alternatively, in order to compute weather-based covariables, based on daily weather data, the user can set the *compute_climatic_ECs* argument to TRUE, and two possibilities are given. The first one is to provide raw daily weather data (with the *raw_weather_data* argument), which will undergo a quality control with the generation of an output file with flagged values. The second possibility, if the user does not have weather data available from measurements (e.g. from an in-field weather station), is the retrieval of daily weather records from the NASA’s Prediction of Worldwide Energy Resources (NASA POWER) database (https://power.larc.nasa.gov/), using the package nasapower (Sparks 2018). Spatiotemporal information contained in the *info_environments* argument is required. Note that the function also checks which environments are characterized by in-field weather data in the *raw_weather_data* argument, in order to retrieve satellite-based weather data for the remaining environments without in-field weather stations. An overview of the pipeline is provided in Fig. 1.

Some covariates are additionally computed, based on the daily weather data, such as vapor pressure deficit or the reference evapotranspiration using the Penman-Monteith (FAO-56) equation. The aggregation of daily information into day-interval-based values is also carried out within this function. Four methods are available and should be specified with the argument *method_ECs_intervals*: (1) default: use of a definite number of intervals across all environments (i.e. the window length varies according to the duration of the growing season); (2) use of day-windows of fixed length (i.e. each window spans a given number of days, which remains identical across environments), that can be adjusted by the user; (3) use of specific day intervals according to each environment provided by the user, which should correspond to observed or assumed relevant phenological intervals; and (4) based on the estimated crop growth stage within each environment using accumulated growing degree-days in degrees Celsius.

Besides weather-based information, soil characterization for each environment can also be provided given the *soil_variables* argument. The output of *create_METData()* is a list object of class *METData*, required as input for all other functionalities of the package.

Box 2. Integration of input data in a METData list object

***Case 1*: ECs directly provided by the user**

> library(learnMET)

> data(geno_indica)

> data(map_indica)

> data(pheno_indica)

> data(info_environments_indica)

> data(env_data_indica)

> METdata_indica <- create_METData(

geno = geno_indica,

map = map_indica,

pheno = pheno_indica,

climate_variables = climate_variables_indica,

info_environments = info_environments_indica,

compute_climatic_ECs = FALSE,

path_to_save =“/learnMET_analyses/indica”)

***Case 2*: daily climate data automatically retrieved and ECs calculated via the package**

> data(geno_G2F)

> data(pheno_G2F)

> data(map_G2F)

> data(info_environments_G2F)

> data(soil_G2F)

> METdata_g2f <- create_METData(

geno = geno_G2F,

pheno = pheno_G2F,

map = map_G2F,

climate_variables = NULL,

raw_weather_data = NULL,

compute_climatic_ECs = TRUE,

info_environments = info_environments_G2F,

soil_variables = soil_G2F,

path_to_save =“/learnMET_analyses/G2F”)

Note: code example to use in-field daily weather data provided at https://cjubin.github.io/learnMET/articles/vignette_getweatherdata.html

#### Machine learning-based models implemented

Different machine learning-based regression methods are provided as S3 classes in an object-oriented programming style. These methods are called within the pipeline of the *predict_trait_MET_cv()* function, that is presented in the following section. In particular, the XGBoost gradient boosting library (Chen and Guestrin 2016), the Random Forest algorithm (Breiman 2001), stacked ensemble models with Lasso regularization as meta-learners (Van der Laan *et al.* 2007), and multilayer perceptrons (MLP) using Keras (Chollet *et al.* 2015) are implemented as prediction methods. In this section, we briefly present how these machine learning algorithms work.

Gradient-boosted decision trees (GBDT) can be seen as an additive regression model, where the final model is an ensemble of weak learners (i.e. a regression tree in this case), in which each base learner is fitted in a forward sequential manner (Friedman 2001). Considering a certain loss function (e.g. mean-squared error for regression), a new tree is fitted to the residuals of the prior model (i.e. an ensemble of trees) to minimize this loss function. Then, the previous model is subsequently updated with the current model. From this definition, it becomes clear that GBDT and Random Forest models strongly differ from each other, since for GBDT, trees are built conditional on past trees, and the trees contribute unequally to the final model (Kuhn *et al.* 2013).

In contrast, in Random Forest algorithms, trees are created independently from each other, and results from each tree are only combined at the end of the process. The concept of GBDT was originally developed by Friedman (2001). In *learnMET*, a set of prediction models, denoted *xgb_reg* and *rf_reg*, is proposed that use the XGBoost algorithm or the Random Forest algorithm, respectively, with different input variables.

An MLP consists of one input layer, one or more hidden layers, and one output layer. Each layer, with the exception of the final output layer, includes a bias neuron (i.e. a constant value that acts like the intercept in a linear equation and is used to adjust the output) and is fully connected to the next layer. Here, the first hidden layer receives the marker genotypes and the ECs as input, computes a weighted linear summation of these inputs (i.e. $z=W^{⊺}\cdot X+b$, where *X* represent the input features, $W^{⊺}$ the vector of weights, and *b* the bias), and transforms the latter with a nonlinear activation function f(z), yielding the output of the given neuron. In the next hidden layers, each neuron (also named node) in one layer connects with a given weight to each neuron in the consecutive layer. The last hidden layer is generally connected with a linear function to the output layer that consists of a single node. In MLP, learning is done via backpropagation: the network makes a prediction for each training instance, calculates the error associated with this prediction, estimates the error contribution from each connection at each hidden layer by iterating backward from the last layer (reverse pass), and finally changes the connection weights to decrease this error, usually using gradient descent step (Géron 2019). For more details about deep learning methods in genomic prediction, we refer to the review written by Pérez-Enciso and Zingaretti (2019). In *learnMET*, a set of prediction models named *DL_reg*, are proposed that apply MLP models with different input variables.

Stacked models can be understood as an ensemble method that exploits the capabilities of many well-working models (called base learners) on a classification or regression task. The theoretical background of this method was originally proposed by Breiman (1996), and further developed by Van der Laan *et al.* (2007). In the first step, different individual base learners are fitted to the same training set resamples (typically generated via CV), and potentially using different sets of predictor variables or different hyperparameter settings. Then, the predictions of the base learners are used as input to predict the output by fitting a regularization method, such as Lasso, on the cross-validated predictions. Hence, the final model has learned how to combine the first-level predictions of the base learners, and this stacked ensemble is expected to achieve similar or better results than any of the base learners (Van der Laan *et al.* 2007). This implies also that some weak learners, trained in the first stage, are generally excluded by variable selection from the resulting ensemble model if their predictions are highly correlated with other models, or irrelevant for predicting the trait of interest. In *learnMET*, prediction models named *stacking_reg* apply stacked ensemble models with different base learners and input variables. For instance, *stacking_reg_3* combines a support vector machine regression model fitted to the ECs, an elastic net model fitted to the SNPs data, and a XGBoost model using as features the 40 genomic-based PCs and the ECs. The stacked model was designed to embrace individual learners as diverse as possible, in order to improve the likelihood that the predictions of the different models are different from each other, and that the meta learning algorithm really benefits from combining these first-level predictions. Regularized regression methods are widely used for genomic selection (Zou and Hastie 2005; de los Campos *et al.* 2013), thus our choice to incorporate Elastic Net as an individual learner to estimate the SNPs effects.

#### Step 2: model evaluation through cross-validation

The second function in a typical workflow is *predict_trait_MET_cv()* (Box 3). The goal of this function is to assess a given prediction method with a specific CV scenario that mimic concrete plant breeding situations.

When *predict_trait_MET_cv()* is executed, a list of training/test splits is constructed according to the CV scheme chosen by the user. Each training set in each sub-element of this list is processed (e.g. standardization and removal of predictors with null variance, feature extraction based on principal component analysis), and the corresponding test set is processed using the same transformations. Performance metrics are computed on the test set, such as the Pearson correlation between predicted and observed phenotypic values (always calculated within the same environment, regardless of how the test sets are defined according to the different CV schemes), and the root mean square error. Analyses are fully reproducible given that seed and tuned hyperparameters are stored with the output of *predict_trait_MET_cv()*. Note that, if one wants to compare models using the same CV partitions, specifying the seed and modifying the model would be sufficient.

The function applies a nested CV to obtain an unbiased generalization performance estimate. After splitting the complete dataset using an outer CV partition (based on either CV1, CV2, CV0, or CV00 prediction problems), an inner CV scheme is applied to the outer training dataset for optimization of hyperparameters. Subsequently, the best hyperparameters are selected and used to train the model using all training data. Model performance is then evaluated based on the predictions of the unseen test data using this trained model. This procedure is repeated for each training-test partition of the outer CV assignments. Table 1 shows the different arguments that can be adjusted when executing the CV evaluation.

**Table 1.** Description of the main arguments used with the function predict_trait_MET_cv().

Function argument	Description
METData	An object created by the initial function of the package create_METData().
trait	Name of the trait to predict.
prediction_method	String to name the trait to predict.
lat_lon_included	Logical to use longitude and latitude as predictor variables. FALSE by default.
yr_included	Logical to use yr effect as dummy variable. FALSE by default.
cv_type	String indicating the CV scheme to use among “cv0” (prediction of genotypes in new environments), “cv00” (prediction of new genotypes in new environments), “cv1” (prediction of new genotypes), or “cv2” (prediction of incomplete field trials). Default is “cv0.”
cv0_type	String indicating the type of cv0 scenario, among “leave-one-environment-out”, “leave-one-site-out”, “leave-one-yr-out”, and “forward-prediction.” Default is “leave-one-environment-out.”
nb_folds_cv1	Integer for the number of folds to use in the cv1 scheme, if selected.
repeats_cv1	Integer for the number of repeats in the cv1 scheme, if selected.
nb_folds_cv2	Integer for the number of folds to use in the cv2 scheme, if selected.
repeats_cv2	Integer for the number of repeats in the cv2 scheme, if selected.
include_env_predictors	Logical to indicate if ECs should be used in predictions. TRUE by default.
list_env_predictors	Vector of character strings with the names of the environmental predictors which should be used in predictions. NULL by default, which means that all environmental predictor variables are used.
seed	Integer with the seed value. Default is NULL, which implies that a random seed is generated, used in the other stages of the pipeline, and given as output for reproducibility.
save_processing	Logical to save the processing steps used to build the model in a RDS file. Default is FALSE.
path_folder	String to indicate the full path where the RDS file with results and plots generated during the analysis should be saved.
num_pcs	Optional argument. Integer to indicate the number of PCs to derive from the genotype matrix or from the genomic relationship matrix (encouraged to speed up CV with large datasets).
save_model	Logical indicating whether the fitted model for each training-test partition should be saved. Default is FALSE.

Note that the classes we developed for preprocessing data and for fitting machine learning-based methods use functions from the tidymodels collection of R packages for machine learning (Kuhn and Wickham 2020), such as Bayesian optimization to tune hyperparameters (function *tune_bayes()*) or the package *stacks*. For models based on XGBoost, the number of boosting iterations, the learning rate, and the depth of trees represent important hyperparameters that are automatically tuned. Ranges of hyperparameter values are predefined based on expert knowledge. Bayesian optimization techniques use a surrogate model of the objective function in order to select better hyperparameter combinations based on past results (Shahriari *et al.* 2016). As more combinations are assessed, more data become available from which this surrogate model can learn to sample new combinations from the hyperparameter space that are more likely to yield an improvement. This technique allows a reduction of the number of model settings tested during the hyperparameter tuning.

Box 3. Evaluation of a prediction method using a CV scheme (i.e. METData object with phenotypic data)

> res_cv0_indica <- predict_trait_MET_cv(

METData = METdata_indica,

trait =“GC”,

prediction_method =“xgb_reg_1”,

cv_type =“cv0”,

cv0_type =“leave-one-year-out”,

seed = 100,

path_folder =“/project1/indica_cv_res/cv0”)

#### Extracting evaluation metrics from the output

Once a model has been evaluated with a CV scheme, various results can be extracted from the returned object, as shown in Box 4, and plots for visualization of results are also saved in the *path_folder*.

Box 4. Extraction of results from returned object of class *met_cv*

# Extract predictions for each test set in the CV scheme:

> pred_2010 <- res_cv0_indica$list_results_cv[[1]]$prediction_df

> pred_2011 <- res_cv0_indica$list_results_cv[[2]]$prediction_df

> pred_2012 <- res_cv0_indica$list_results_cv[[3]]$prediction_df

# The length of the list_results_cv sub-element is equal to the number of train/test sets partitions.

# Extract Pearson correlation between predicted and observed values for 2010:

> cor_2010 <- res_cv0_indica$list_results_cv[[1]]$cor_pred_obs

# Extract root mean square error between predicted and observed values for 2011:

> rmse_2011 <- res_cv0_indica$list_results_cv[[2]]$rmse_pred_obs

# Get the seed used:

> seed <- res_cv0_indica$seed_used

#### Step 3: prediction of performance for a new test set

The third module in the package aims at implementing predictions for unobserved configurations of genotypic and environmental predictors using the function *predict_trait_MET()* (Box 5). The user needs to provide a table of genotype IDs (e.g. name of new varieties) with their growing environments (i.e. year and location) using the argument *pheno* in the function *create_METData()*. Genotypic data of the selection candidates to test within this test set should all be provided using the *geno* argument. Regarding characterization of new environments, the user can either provide a table of environments, with longitude, latitude, and growing season dates, or can directly provide a table of ECs that should be consistent with the ECs provided for the training set. Environmental variables for the unobserved test set should be provided or computed with the same aggregation method (i.e. same *method_ECs_intervals*) as for the training set. To build an appropriate model with learning parameters, able to generalize well on new data, a hyperparameter optimization with CV is conducted on the entire training dataset when using the function *predict_trait_MET()*.

This function can potentially be applied to harness historical weather data and to obtain predictions across multiple years at a set of given locations (de Los Campos *et al.* 2020), or to conjecture about the best selection candidates to assess in field trials at specific locations. However, we emphasize the importance of both environmental and genetic similarity between training and test sets. If the selection candidates within the test set are not strongly genetically related to the genotypes included in the training set, or if the climatic conditions experienced in the test set differ too much from the feature space covered within the training set, the prediction results might not be trustworthy for decision making.

The function *analysis_predictions_best_genotypes()* takes directly the output of *predict_trait_MET()* and can be used to visualize the predicted yield of the best performing genotypes at each of the locations across years included in the test set.

Box 5. Prediction of new observations using a training set and a test set (i.e. phenotypic data not required)

# Create a training set composed of years 2014, 2015 and 2016:

> METdata_G2F_training <-

create_METData(

geno = geno_G2F,

pheno = pheno_G2F[pheno_G2F$year %in% c(2014,2015,2016),],

map = map_G2F,

climate_variables = NULL,

compute_climatic_ECs = TRUE,

et0 = T, # Possibility to calculate reference evapotranspiration with the package (if TRUE, elevation data should be preferably added as a column in info_environments)

info_environments = info_environments_G2F[info_environments_G2F$year %in% c(2014,2015,2016),],

soil_variables = soil_G2F[soil_G2F$year %in% c(2014,2015,2016),],

path_to_save =“/project1/g2f_trainingset”) # path where daily weather data and plots are saved

# Create a prediction set (same default method to compute ECs as above):

> METdata_G2F_new <-

create_METData(

geno = geno_G2F,

pheno = as.data.frame(pheno_G2F[pheno_G2F$year %in% 2017 , ] % >% dplyr::select(-pltht, -yld_bu_ac, -earht)),

map = map_G2F,

et0 = T,

climate_variables = NULL,

compute_climatic_ECs = TRUE,

info_environments = info_environments_G2F[info_environments_G2F$year %in% 2017 , ],

soil_variables = soil_G2F[soil_G2F$year %in% 2017 , ],

path_to_save =“/project1/g2f_testset”,

as_test_set = T) # in order to provide only predictor variables (no phenotypic data for the test set available) in *pheno* argument.

# Fitting the model to the training set and predicting the test set

> results_list <- predict_trait_MET(

METData_training = METdata_G2F_training,

METData_new = METdata_G2F_new,

trait =“yld_bu_ac”,

prediction_method =“xgb_reg_1”,

use_selected_markers = F,

save_model = TRUE,

# save_model set to TRUE in order to retrieve subsequently variable importance

lat_lon_included = F,

year_included = F,

num_pcs = 200,

include_env_predictors = T,

seed = 100,

path_folder =“/project1/g2f_results_year_2017”

)

#### Interpreting ML models

Compared to parametric models, ML techniques are often considered as black-boxes implementations that complicate the task of understanding the importance of different factors (genetic, environmental, management, or their respective interactions) driving the phenotypic response. Therefore, various methods have recently been proposed to aid the understanding and interpretation of the output of ML models. Among these techniques, some are model-specific techniques (Molnar 2022), in the sense that they are only appropriate for certain types of algorithms. For instance, the Gini importance or the gain-based feature importance measures can only be applied for tree-based methods (e.g. decision trees, Random Forests, gradient-boosted trees), since it calculates how much a predictor variable can reduce the sum of squared errors in the child nodes, compared to the parent node, across all splits for which this given predictor was used. Feature importances are in this case scaled between 0 and 100.

Other model-agnostic interpretation techniques have been developed, that provide the advantage of being independent from the original machine learning algorithm applied, thereby allowing straightforward comparisons across models (Molnar 2022). After shuffling the values of a given predictor variable, the value of the loss function (e.g. root mean square error in regression problems), estimated using the predictions of the shuffled data and the observed values, can be used to obtain an estimate of the permutation-based variable importance. Fisher *et al.* (2019) formally defined the permutation importance for a variable *j* as follows: $vip_{\text{diff}}^{j}=L(y,\;\hat{f}(X_{\text{permuted}}))-L(y,\;\hat{f}(X_{\text{original}}))$, where $L(y,\;\hat{f}(X))$ is the loss function evaluating the performance of the model, *X*_original_ is the original matrix of predictor variables, and *X*_permuted_ is the matrix obtained after permuting the variable *j* in *X*_original_. The reason behind this approach is that, if a predictor contributes strongly to a model’s predictions, shuffling its values will result in increased error estimates. On the other hand, if the variable is irrelevant for the fitted model, it should not affect the prediction error. It is recommended to repeat the permutation process to obtain a more reliable average estimate of the variable importance (Fisher *et al.* 2019; Molnar 2022). Another interesting aspect of permutation-based variable importance is the possibility to calculate it using either the training or the unused test set. Computing variable importance using unseen data is useful to evaluate whether the explanatory variables, identified as relevant for prediction during model training, are truly important to deliver accurate predictions, and whether the model does not overfit. However, in the latter case, one needs to ensure that the training and test set are sufficiently related. New data might behave very differently from the data used for training without implying that the trained model is fundamentally wrong. The function *variable_importance_split()* enables retrieving variable importance, either with a model-specific method (via the package vip proposed by Greenwell *et al.* 2020), when available, or based on a permutation-based method (argument *type*, see Box 6), and the calculation is made by default using the training set, but can be achieved for the test set by setting the argument *unseen_data* to TRUE.

Box 6. Retrieving variable importance using the fitted model and the training data

> fitted_split <- results_list$list_results[[1]]

# Model-specific: variable importance based on the gain as importance metric from the XGBoost model (via vip package)

> variable_importance <- variable_importance_split(

object = fitted_split,

path_plot =“/project1/variable_imp_trset”,

type =“model_specific”)

# Model-agnostic: variable importance based on 10 permutations

> variable_importance <- variable_importance_split(

object = fitted_split,

path_plot =“/project1/variable_imp_trset”,

type =“model_agnostic”,

permutations = 10)

# Model-agnostic: accumulated local effects plot

> ALE_plot_split(fitted_split,

path_plot =“/project1/ale_plots,”

variable =”freq_P_sup10_2”)

Accumulated local effects (ALE) plots, also model agnostic, allow to examine the influence of a given predictor variable on the model prediction, conditional on the predictor value (Apley and Zhu 2020). Compared to partial dependence (PD) plots, they provide the advantage of addressing the bias that emerges when features are correlated. While predictions are computed over the marginal distribution of predictor variables in the case of PD plots (i.e. meaning that predictions of unrealistic instances are considered), ALE plots offer a solution to this issue by considering the conditional distribution, thus avoiding to use predictions of unrealistic training observations. To build an ALE plot, the range of the explanatory variable is first split into equally sized small windows, such as quantiles. For each window, the ALE method only considers observations that show for this feature a value falling within the interval. Then, it computes model predictions for the upper limit and for the lower limit of the interval for these data instances, and calculates the difference in predictions. The changes of predictions are averaged within each interval, which allows to block the impact of other features. These average effects are then accumulated across all intervals and centered at 0. The function *ALE_plot_split()* yields the ALE plot for a given predictor variable. An example is provided in Box 6.

## Results and discussion

To illustrate the use of learnMET with METs datasets, we provide here two example pipelines, both of which are available in the official package documentation. The first one demonstrates an implementation that requires no user-provided weather data, while the second pipeline shows prediction results obtained based on user-provided environmental data.

### Retrieving meteorological data from NASA POWER database for each environment

When running the commands for step 1 (Box 1, Case 2) on the maize dataset, a set of weather-based variables (see documentation of the package) is automatically calculated using weather data retrieved from the NASA POWER database. By default, the method used to compute ECs uses a fixed number of day-windows (10) that span the complete growing season within each environment. This optional argument can be modified via the argument *method_ECs_intervals* (detailed information about the different methods can be found at https://cjubin.github.io/learnMET/reference/get_ECs.html). The function *summary()* provides a quick overview of the elements stored and collected in this first step of the pipeline (Box 7).

Box 7. Summary method for class METData

> summary(METdata_g2f)

**Fig. 2.**
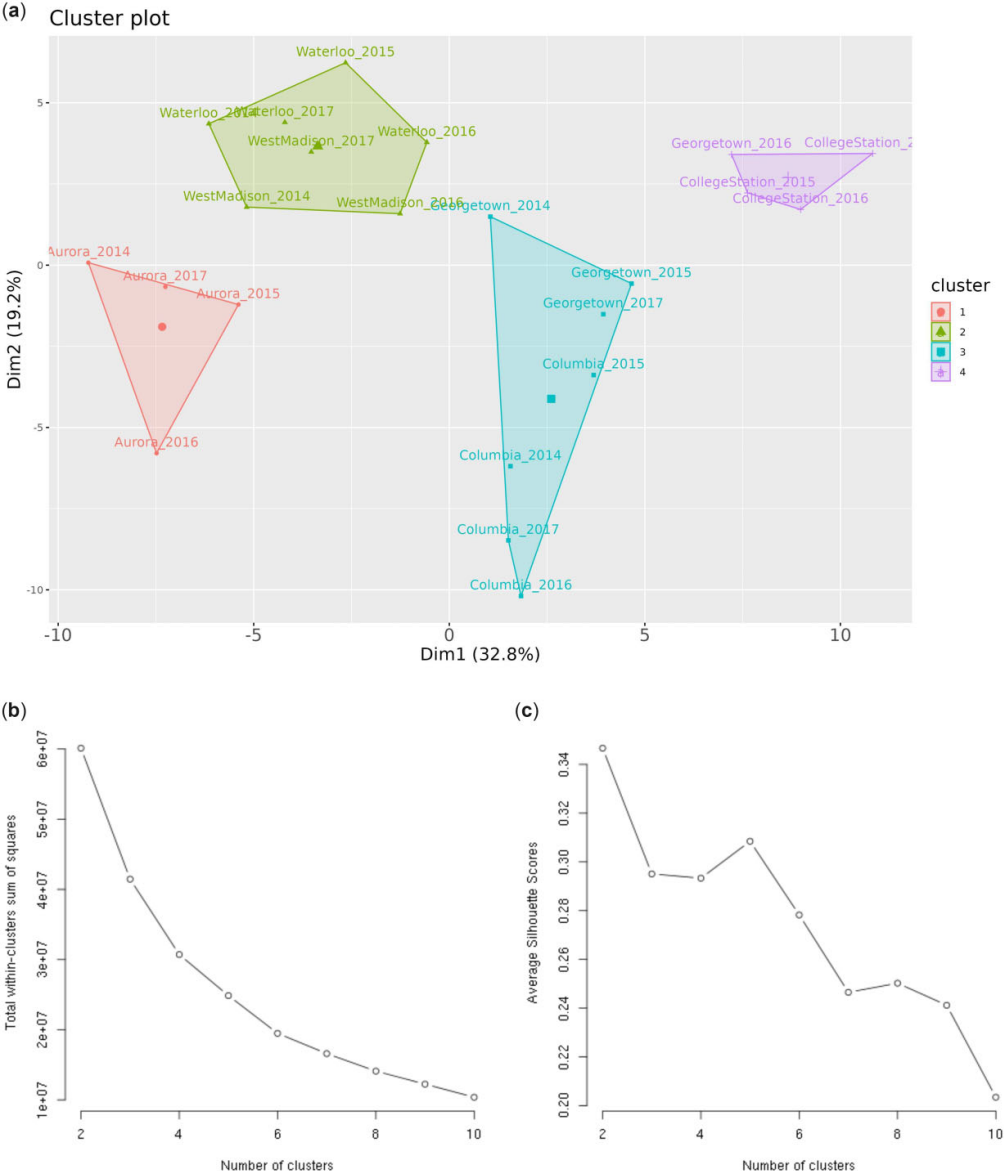
Output results from the *create_METData()* function. a) Cluster analysis using *K*-means algorithm (*K* = 4) to identify groups of similar environments based on environmental data. b) Total within-cluster sum of squares as a function of the number of clusters. c) Average Silhouette score as a function of the number of clusters. These methods can help users decide on the optimal number of clusters. Data used here are a subset of the Genomes to Fields maize dataset (AlKhalifah *et al.* 2018; McFarland *et al.* 2020). Weather data were retrieved from NASA POWER database via the package nasapower Sparks (2018). Plots are saved in the directory provided in the *path_to_save* argument.

Clustering analyses, that can help to identify groups of environments with similar climatic conditions and to identify outliers, were generated based on (a) only climate data; (b) only soil data (if available); and (c) all environmental variables together, for a range of values for *K* = 2 to 10 clusters (Fig. 2).

### Benchmarking two prediction methods from *learnMET* and a linear reaction norm model

Phenotypic traits were predicted by the reaction norm model proposed by Jarquín *et al.* (2014), thereafter denoted as G-W-G × W, that account for the random linear effects of the molecular markers (G), of the environmental covariates (W), and of the interaction term (G × W), under the following assumptions:

$$y_{ij}=\mu +g_{i}+w_{j}+gw_{ij}+\varepsilon_{ij},$$

with $g∼N(0,G\sigma_{g}^{2})$, where G=XX′/p (with *p* being the number of SNPs and *X* the scaled and centered marker matrix), $w∼N(0,\Omega \sigma_{w}^{2})$, where Ω=WW′/q (with *q* being the number of ECs and *W* the scaled and centered matrix that contains the ECs), $gw∼N(0,[Z_{g}GZ_{g}^{\prime }]\circ \Omega \sigma_{gw}^{2}$) where ° denotes the Hadamard product (cell by cell product), $\varepsilon_{ij}\overset{\text{IID}}{∼}N(0,\sigma_{\varepsilon }^{2})$.

For additional details about the benchmark model, we refer to the original publication of Jarquín *et al.* (2014). We implemented this model using BGLR (Pérez and de Los Campos 2014), for which the MCMC algorithm was run for 20,000 iterations and the first 2,000 iterations were removed as burn-in using a thinning equal to 5.

Two prediction models proposed in *learnMET* were tested: (1) *xgb_reg_1*, which is an XGBoost model that uses a certain number of principal components (PCs) derived from the marker matrix and ECs, as features and (2) *stacking_reg_3*. Although computationally more expensive than parametric methods, we paid attention to reasonable computational time (e.g. maximum of 13.3 hours to fit *stacking_reg_3* model to *n* = 4,587 training instances with 10 CPUs).

We conducted a forward CV0 CV scheme, meaning that future years were predicted when using only past years as the training set. For the rice datasets, at least two years of data were used to introduce variation in the EC matrix characterizing the training set (only one location was tested each year). Year, location or year-location effects were not incorporated in any of the linear and machine learning models, because we focused our evaluation on how the different models could efficiently capture the effects of SNPs and ECs, and of SNP × EC interaction effects.

**Fig. 3.**
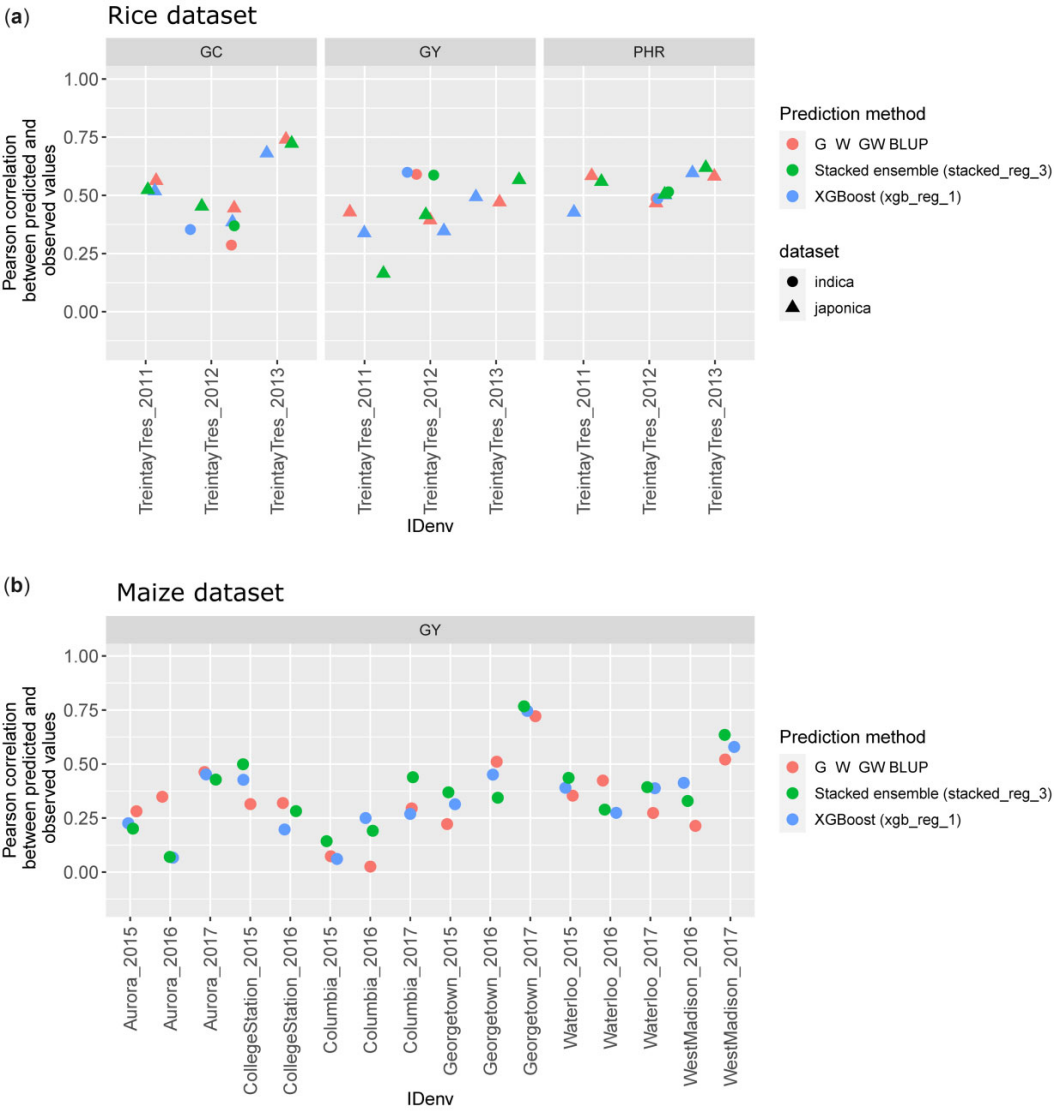
Correlations between predicted and observed values for a forward prediction scenario using two machine learning models and a linear reaction norm approach. a) Three traits predicted for two rice populations. Each year is predicted based on at least two past years of phenotypic data (one single location). b) Grain yield predicted for the G2F dataset. GC (rice data), percentage of chalky kernels; GY (rice data), grain yield (kg/ha); PHR (rice data), percentage of head rice recovery; GY (G2F), bushels per acre.

**Fig. 4.**
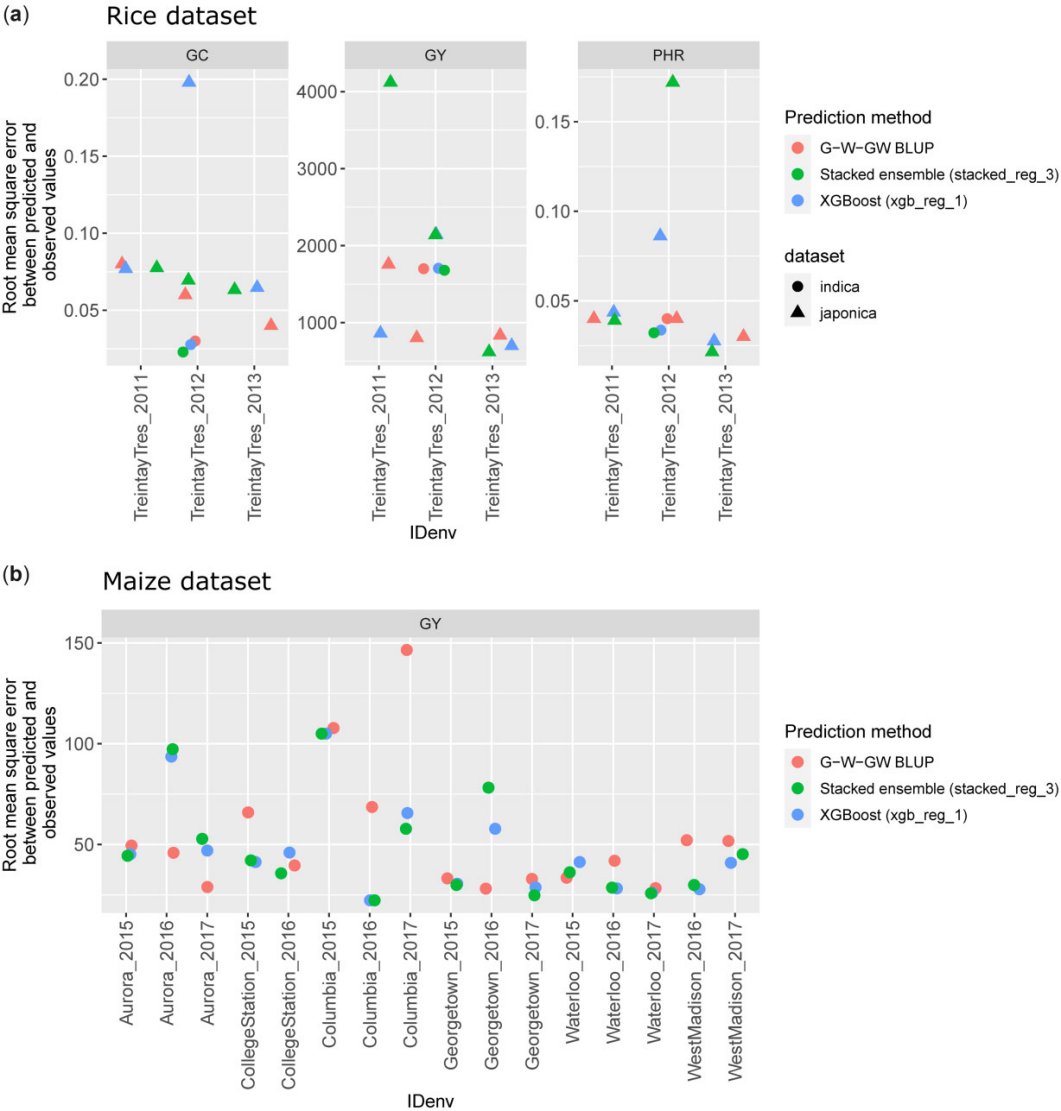
Root mean square error between predicted and observed values for a forward prediction scenario using two machine learning models and a linear reaction norm approach. a) Three traits predicted for two rice populations. Each year is predicted based on at least two past years of phenotypic data (one single location). b) Grain yield predicted for the G2F dataset. GC (rice data), percentage of chalky kernels; GY (rice data), grain yield (kg/ha); PHR (rice data), percentage of head rice recovery; GY (G2F), bushels per acre.

Results from the benchmarking approach are presented in Figs. 3 and 4. We have observed that the machine learning models are competitive with the linear reaction norm approach and tend to outperform it, albeit not consistently, as the training set size increases. Applied to small training set sizes, sophisticated prediction models are likely not able to capture informative patterns related to SNP × EC interactions, and linear models perform better. Similarly, the root mean square error was generally reduced with the machine learning methods as the training set increased (Fig. 4). Machine learning also performed better with the G2F data that integrated multiple locations per year and was therefore larger and probably more relevant to learn G × E patterns than with the rice dataset. Therefore, we encourage users to first evaluate whether their datasets are sufficiently large to leverage the potential of the advanced techniques proposed in this package and whether the latter provide satisfying predictive abilities in CV settings.

### Model interpretation from a gradient-boosted model fitted to the maize dataset

**Fig. 5.**
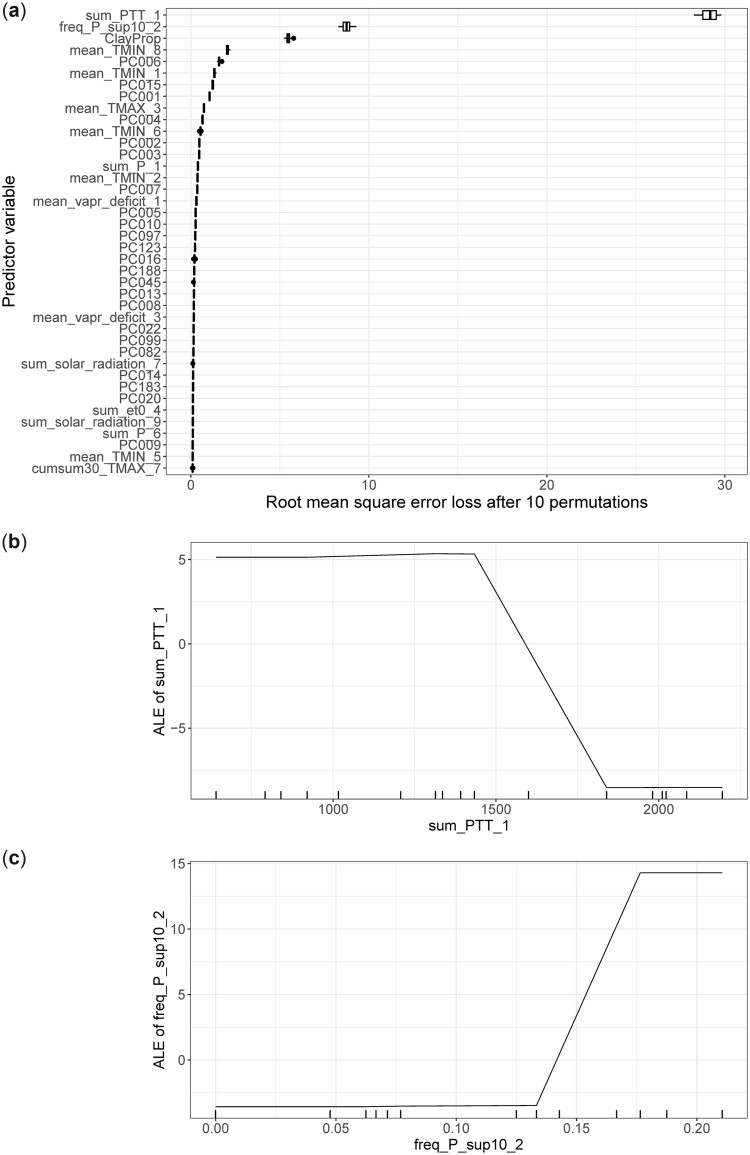
Model interpretation methods applied on the model fitted to a subset of the G2F dataset from years 2014 to 2016 (17 environments included) with *xgb_reg_1* for the trait grain yield. a) Model-agnostic variable importance using 10 permutations. The top 40 most important predictor variables are displayed, and the table containing results across all permutations for all variables is returned. ALE plots for (b) sum of photothermal time during the 1st day-interval of the growing season, and (c) the frequency of days with an amount of precipitation above 10 mm during the 2nd day-interval of the growing season. Tick marks indicate the unique values observed for the given covariate in the training set.

Figure 5a illustrates the permutation-based approach on the maize dataset, and Fig. 5, b and c describe how two environmental variables (sum of photothermal time and frequency of rainfall) influence the average prediction of maize grain yield using ALE plots. We should stress that the size of the dataset employed here is likely too small to make real inferences about the relationship between the predictor variables and the outcome (sharp drops observed at some feature values). Our goal here is essentially to illustrate how these functions can be used to gain insights into a model’s predictions using the package.

## Concluding remarks and future developments

*learnMET* was developed to make the integration of complex datasets, originating from various data sources, user-friendly. The package provides flexibility at various levels: (1) regarding the use of weather data, with the possibility to provide on-site weather station data, or to retrieve external weather data, or a mix of both if on-site data are only partially available; (2) regarding how time intervals for aggregation of daily weather data are defined; (3) regarding the diversity of nonlinear machine learning models proposed; (4) regarding options to provide manually specified subsets of predictor variables (for instance, for environmental features via the argument *list_env_predictors* in *predict_trait_MET_cv()*).

To allow analyses on larger datasets, future developments of the package should include parallel processing to improve the scalability of the package and to best harness high performance computing resources. Improvements and extensions of stacked models and deep learning models are also intended, as we did not investigate in-depth the network architecture (e.g. number of nodes per layer, type of activation function, type of optimizer), nor other types of deep learning models that might perform better (e.g. convolutional neural networks). Finally, the package could be extended to allow genotype-specific ECs, because the timing of developmental stages differs across genotypes (e.g. due to variability in earliness) and should ideally be taken into account.

## Data Availability

The software is available on GitHub at https://github.com/cjubin/learnMET. Documentation and vignettes are provided at https://cjubin.github.io/learnMET/. All scripts used to obtain the results presented in this article can be found on GitHub at https://github.com/cjubin/learnMET/tree/main/scripts\_publication.
